# A Static Phase-Bank CNN Demodulator for Rolling-Shutter Optical Camera Communication in Intra-Satellite Links

**DOI:** 10.3390/s26206641

**Published:** 2026-10-20

**Authors:** Jaime Aranda Cubillo, Jose Rabadan, Rafael Perez-Jimenez

**Affiliations:** Institute for Technological Development and Innovation in Communications, Universidad de Las Palmas de Gran Canaria, 35001 Las Palmas de Gran Canaria, Spain; jose.rabadan@ulpgc.es (J.R.); rafael.perez@ulpgc.es (R.P.-J.)

**Keywords:** optical camera communication, rolling shutter, intra-satellite communication, visible light communication, convolutional neural network, learned receiver, demodulation

## Abstract

This work proposes and evaluates a convolutional neural network (CNN) for demodulating LED-transmitted on–off keying signals captured by a rolling-shutter camera, motivated by short-range intra-satellite optical communication. The proposed Static Phase Bank predicts payload bits and their validity directly from camera images, without an external intensity threshold or payload-start detector. A central objective is to investigate training with synthetic frames. Real recordings are used to calibrate the appearance of generated rolling-shutter images, whose payload locations and transmitted bits are known by construction. The CNN is then trained exclusively on these synthetic frames and evaluated on real recordings at 30 lx, without fine-tuning. It achieves a frame-local reference-based BER of $1.79\times 10^{-3}$ and correctly recovers all labelled payloads and slot-validity decisions in 97.6% of test frames. A separate experiment demonstrates that one CNN trained jointly on 42 lx and 4 lx recordings can demodulate both conditions using the same network parameters. Real-data results are measured against reviewed proxy labels and do not establish general robustness or an accuracy advantage over conventional receivers. Nevertheless, the study demonstrates two practical contributions of the CNN approach: training on synthetic frames while retaining useful demodulation performance on real images, and incorporating multiple illumination conditions into a single receiver without condition-dependent model switching or threshold adjustment.

## 1. Introduction

### 1.1. Intra-Satellite Application and Motivation

Short-range optical links are being investigated as an alternative to selected harnessed connections in small spacecraft, where wiring adds mass, occupies internal volume, and complicates integration [1,2]. Optical camera communication (OCC) uses an image sensor to obtain both spatial information and intensity observations [3]. With rolling-shutter acquisition, successive image rows sample different time intervals, allowing an intensity-modulated LED to produce data-bearing stripes above the camera frame rate [4,5,6].

The present work uses a laboratory demonstrator as a ground-based experimental analogue of the intended OCC4SAT-inspired intra-satellite link. Dark-room acquisition is deliberately chosen to approximate the low-ambient-light conditions expected inside the enclosed satellite, where the communication LED is the intended optical source. The laboratory link uses a short, direct optical path between the LED and camera. Because camera acquisition and packet transmission are not synchronized, payloads appear at different vertical positions within the captured images. The CNN processes a fixed-size LED ROI and predicts up to two payload bytes together with their validity.

Conventional OOK demodulation combines header detection, intensity thresholding, and bit-decision rules. A fixed threshold is effective under the controlled illumination conditions considered here, while automatic or adaptive threshold selection provides another approach to determining the decision level. A CNN offers a different strategy: learning to recover payload bits and determine their validity directly from camera images. This motivates investigating whether receiver behaviour can be established through training rather than condition-specific intensity-threshold selection. In particular, calibrated synthetic images can provide labelled training examples, and recordings from multiple LED intensity conditions can be incorporated into one training set. The objective is therefore to evaluate whether synthetic-only training produces useful demodulation performance on real images, and whether one jointly trained network can operate at both measured intensity levels without changing its weights or adjusting an external threshold.

Capturing real images and associating their visible payloads with transmitted bytes requires an acquisition and labelling procedure. Once a synthetic image generator has been calibrated from real recordings, additional examples can instead be generated with packet positions and payload bits known by construction. This permits controlled variation of packet contents and positions without physically recording and associating every training example. Real-image evaluation then tests whether the learned decoding transfers beyond the generated images.

All experiments use the same input image dimensions and output format: two possible payload bytes and their validity indicators. In the joint-training experiment, the varied condition is the communication LED drive, not external ambient illumination: changing the drive changes the received image intensity even in a dark room. High- and low-drive examples are therefore used to investigate operation with the same weights and preprocessing at both levels.

### 1.2. Related Work and Training-Data Requirements

Classical RS-OCC receivers combine row-intensity extraction, preamble detection, thresholding, symbol decisions, and packet repetition or reconstruction [5,7]. Under a stable dark-room configuration, a fixed threshold can be both accurate and substantially cheaper than a CNN. Automatic threshold selection is a useful comparison because it changes the decision level from the observed image, but does not by itself remove symbol-timing or packet-location assumptions [8].

Learning-based physical-layer processing has been studied both as an alternative to individual receiver blocks and as joint transmitter–receiver design [9]. Lee et al. review deep-learning approaches for optical wireless systems and investigate an image-sensor communication framework [10]. Ma et al. demonstrate machine-learning demodulation using a measured VLC dataset, including a CNN-based method [11]. These works provide a broader background for learning-based optical reception, but their signal representations and tasks differ from the full-ROI rolling-shutter byte-and-validity prediction studied here.

Learned OCC processing predates this study. Liu et al. used a two-dimensional CNN and XOR-based data-loss compensation in an RGB-LED OCC system reporting 47kbit/s [12]. Lin et al. proposed pixel-per-symbol labeling and resampling for neural PAM4 demodulation [13]. Jurado-Verdu et al. developed a convolutional autoencoder to mitigate exposure-related intersymbol interference and noise using synthetic training images [14]; their later work estimates signal clock and exposure [15]. Background removal and neural processing have also been applied to display-panel rolling-shutter links [16]. These studies address higher-rate transmission, equalization, symbol-level classification, or acquisition-parameter estimation. Their results cannot be ranked directly against this paper because their modulation, images, packet interfaces, and reference protocols differ.

Related event-camera OCC studies have investigated N-pulse modulation [17] and pulse-shaped waveform modulation [18]. They process asynchronous brightness-change events rather than conventional rolling-shutter image frames, so their receiver results are not direct accuracy benchmarks for the present CNN.

Learning shifts part of receiver calibration into dataset construction. Diverse labelled measurements can be costly, and labels derived from another receiver inherit that receiver’s selection biases. Generative augmentation and few-shot signal recognition provide methodological context: Ding et al. combine a conditional generative adversarial network with CNN-based jamming-signal recognition [19]. This is an RF classification study, not evidence of OCC performance. Here, physics-informed exposure rendering with empirical camera appearance supplies training images whose packet locations and bytes are known before rendering. The relevant test is not synthetic accuracy alone, but whether a synthetically trained, frozen receiver also demodulates real camera observations. Calibration remains necessary, and prior synthetic OCC studies preclude a claim that synthetic training itself is new.

### 1.3. Research Gap and Contributions

The proposed Static Phase Bank is a fixed-shape, full-ROI CNN for joint frame-local payload and validity inference. It preserves a dense axis of candidate payload starts, derives two latent slot-position score maps, and fuses this evidence into two ordered bytes and their validity. This packet-specific composition enables direct inference without an external intensity threshold or payload-start detector; it is not a new convolutional primitive or the first learned OCC receiver. Its individual components still require controlled ablations.

The principal contribution is to demonstrate two capabilities of the proposed CNN demodulator: learning from synthetic images while retaining useful performance on real recordings, and operating across high- and low-intensity LED conditions with a single set of trained weights. These capabilities are evaluated in two separate experiments. In the first, the CNN is trained exclusively on synthetic frames calibrated from real development recordings and evaluated on real images without fine-tuning. In the second, a separate model with the same architecture is trained jointly on high- and low-illuminance recordings and tested on a separate recording at each level. The jointly trained model uses unchanged weights and image preprocessing at both levels, without condition-dependent model switching or external intensity-threshold adjustment.

These are demonstrated capabilities of the learned receiver, not proof that conventional receivers cannot use simulation for calibration or cannot cover multiple conditions. The fixed-threshold comparisons use separately calibrated levels; a single shared conventional threshold has not been exhaustively tested. The local adaptive comparison also uses one shared parameter setting at both measured illuminance levels. Reviewed real labels also inherit threshold-based selection bias. The evaluation therefore includes conventional and representative-CNN comparisons and resource accounting to delimit the claims rather than assert an unmeasured accuracy advantage. A fixed graph offers a possible route toward edge-camera conversion [20,21], but conversion and deployment have not been demonstrated.

Section 2 defines the link and acquisition setup. Section 3 separates real proxy labels, simulator ground truth, and their dataset roles. Section 4 specifies receivers, training, and metrics. Section 5 presents synthetic-to-real and joint-condition results first, followed by comparison experiments, resources, and remaining validation requirements. Section 6 concludes with the two practical training contributions and their scope.

## 2. System Model and Experimental Setup

The considered link is a laboratory optical camera communication chain motivated by intra-satellite data transfer. It comprises an LED transmitter, a short-range optical path, a rolling-shutter image sensor, ROI extraction, and a packet-compatible demodulator. This section defines the OOK signal and camera observations shared by the real and synthetic datasets. The experiments keep the packet format and ROI interface fixed while examining two training routes: synthetic-only fitting followed by frozen real-image inference, and joint fitting on two measured LED illuminance levels. These routes evaluate how the receiver can be trained; they do not assume that the original dark-room link suffers from threshold errors. Network details and evaluation protocols follow in Section 4.

Figure 1 summarizes the laboratory OCC chain. The STM32-based conditioner and signal board maps the input data to the LED drive signal. The modulated LED is observed through the short-range optical channel by the camera receiver, and the single-board computer extracts the camera evidence for demodulation and packet recovery.

### 2.1. Transmitter and Packet Model

The transmitter emits an on–off keying (OOK) optical waveform. Each transmitted character is encoded using standard seven-bit ASCII and represented as an eight-bit byte by prepending a zero-valued most-significant bit. Let $b_{i}\in \left\{0,1\right\}$ denote the *i*th payload bit in MSB-first transmission order. The payload is therefore $b=[b_{0},\ldots ,b_{7}]$, where $b_{0}=0$ is the leading bit and $\left[b_{1},\ldots ,b_{7}\right]$ contains the seven-bit ASCII character code. This byte representation defines the payload length as eight OOK symbols. Let $h=\left[1,\ldots ,1\right]\in {\left\{0,1\right\}}^{8}$ denote the eight-symbol header. Because each header-plus-payload packet is transmitted twice, the complete transmitted sequence for one character is

(1) $$a=\left[a_{0},\ldots ,a_{31}\right]=\left[h,b,h,b\right],$$

where $a_{k}$ is the *k*th transmitted OOK symbol, h is the synchronization header or preamble, and b is the payload. Each packet copy therefore contains eight consecutive one-valued header symbols followed by the eight-bit ASCII payload, as illustrated in Figure 2. The two copies provide redundancy for asynchronous frame acquisition. Repetition alone is not a proof of alias-free sampling or complete packet recovery in the presence of camera blanking.

The transmitter uses rectangular pulse shaping, so the optical level remains constant throughout each symbol period $T_{\mathrm{sym}}$. During the interval assigned to packet symbol $a_{k}$, the emitted optical power is

(2) $$s\left(t\right)=P_{\mathrm{off}}+\left(P_{\mathrm{on}}-P_{\mathrm{off}}\right)a_{k},\;kT_{\mathrm{sym}}\leq t<\left(k+1\right)T_{\mathrm{sym}},$$

where k∈{0,…,31} and $P_{\mathrm{on}}$ and $P_{\mathrm{off}}$ are the optical levels associated with binary one and zero, respectively. Thus, $a_{k}=1$ holds the LED at $P_{\mathrm{on}}$ for one complete symbol period, whereas $a_{k}=0$ holds it at $P_{\mathrm{off}}$.

Figure 3 shows a representative oscilloscope trace of the emitted OOK waveform, including the repeated header and payload intervals.

### 2.2. Optical Channel and Image Formation

The camera does not observe the emitted optical power s(t) directly. For the fixed line-of-sight laboratory geometry, the optical path is approximated as quasi-static and memoryless over one recording. The irradiance formed at the sensor plane is modeled as

(3) $$E_{\mathrm{rx}}\left(x,y,t\right)=s\left(t\right)h_{\mathrm{opt}}\left(x,y;\phi \right)+E_{\mathrm{amb}}\left(x,y,t\right),$$

where (x,y) denotes sensor-plane coordinates, $h_{\mathrm{opt}}\left(x,y;\phi \right)$ is the spatial optical channel response, and $E_{\mathrm{amb}}\left(x,y,t\right)$ is the ambient irradiance. The channel response combines propagation loss, transmitter–receiver alignment, the LED and diffuser radiation pattern, lens collection, and the spatial footprint formed on the sensor. The parameter vector ϕ describes these optical and geometric conditions, including distance, alignment, and focus. Reflected components that cannot be resolved separately are included in the effective channel response or the ambient term according to whether they vary with the transmitted signal. Camera exposure, electronic gain, sensor noise, clipping, and quantization are applied after this optical model and are therefore represented in the rolling-shutter image-formation model of Section 2.3. This separation follows the usual intensity-modulation/direct-detection OCC abstraction, in which the optical channel acts on the emitted intensity before row-wise exposure and camera readout [4,22].

### 2.3. Rolling-Shutter Sampling

In a rolling-shutter image sensor, consecutive rows begin their exposures at different times. For frame *m* and ROI row r∈{0,…,H−1}, where H=480 is the ROI height, the exposure start is approximated by

(4) $$t_{m,r}=t_{m,0}+rT_{\mathrm{row}},$$

where $t_{m,0}$ is the exposure start of the first ROI row in frame *m* and $T_{\mathrm{row}}$ is the row period, or line time, between consecutive exposure starts. The exposure time $T_{\exp}$ is a separate camera parameter and may be longer than $T_{\mathrm{row}}$.

For color channel d∈{R,G,B}, the resulting RGB pixel value at row *r* and column *w* is approximated as

(5) $$I_{m,d}\left[r,w\right]=C_{d}\left(\gamma_{d}\int_{t_{m,r}}^{t_{m,r}+T_{\exp}}\int_{A_{r,w}}E_{\mathrm{rx}}\left(x,y,t\right)\;dx\;dy\;dt+\beta_{d}+n_{m,r,w,d}\right).$$

Here, $E_{\mathrm{rx}}\left(x,y,t\right)$ is the sensor-plane irradiance given by Equation (3), which is integrated over the exposure interval of row *r* and over the photosensitive pixel area $A_{r,w}$. The factor $\gamma_{d}$ includes the spectral response and effective gain of color channel *d*, $\beta_{d}$ is its offset, and $n_{m,r,w,d}$ represents sensor-domain noise. The operator $C_{d}\left(\cdot \right)$ represents the fixed camera processing, clipping, and quantization used to produce the RGB888 output, that is, an image with three color channels quantized to eight bits each, so that every pixel value satisfies $I_{m,d}\left[r,w\right]\in \left\{0,\ldots ,255\right\}$. The complete RGB image for frame *m* is denoted by $I_{m}$.

Each row therefore contains an integration of the received irradiance over a finite interval rather than an instantaneous sample. When this interval overlaps an OOK symbol boundary, energy from adjacent symbols contributes to the same row. This integration produces softened stripe transitions and small variations in the measured rows per symbol [5,22].

### 2.4. Geometry, Acquisition Settings, and LED Illuminance Levels

The laboratory demonstrator uses a line-of-sight optical path between a modulated LED strip and a Raspberry-Pi-connected camera receiver. Figure 4 shows the complete experimental bench, where the optical source, camera receiver, and embedded processing hardware are mounted on an optical breadboard. The LED strip is placed behind a diffuser, while the receiver camera is mechanically fixed in front of the optical source at a reported LED-to-camera distance of 40 cm. The LED and camera face each other, with nominally aligned optical axes (approximately normal incidence); no angular metrology or tolerance was recorded.

All recordings were acquired in a dark room to approximate the low-ambient-light environment expected for the intended enclosed intra-satellite link. External illumination was negligible, and the modulated LED was the dominant optical source within the ROI. This ground-based experiment substitutes for deployment when evaluating receiver operation under that illumination assumption; it does not reproduce the complete spacecraft environment. No calibrated ambient-illuminance measurement or quantitative match to a flight enclosure is claimed. Changes in LED drive below concern the communication source, not changes in external ambient illumination.

The original session did not deliberately vary geometry, exposure, or ROI position. The LED illumination conditions are specified by the illuminance measured at the receiver plane: 42 lx for the high-level development/test recordings, 4 lx for the low-level development/test recordings, and 30 lx for the synthetic-to-real evaluation. For the high- and low-illuminance recordings, the camera acquisition settings were kept unchanged while the LED drive was varied. The original dark-room condition is not asserted to create a threshold problem.

Table 1 summarizes the configuration used to acquire the RGB ROI dataset. Frames are requested in RGB888 format at 640×480 pixels. The IMX219 sensor used by Raspberry Pi Camera Module v2.1 expresses exposure in whole row periods and does not apply the original 1μs software request. For the configured 640×480 mode, the effective minimum exposure is approximately 75μs, which is the value reported in Table 1. The full-frame capture is reduced only by spatial cropping around the optical footprint; no interpolation or resizing is applied before the CNN. Six captures of 5000 frames were recorded for the final experiment. The original proxy export retained 29,998 frames; subsequent association review retains all 30,000 frames. Five captures form normal-level development and the sixth forms Source-15 evaluation, as detailed in Section 3.4. Additional intensity experiments use the recording-level partitions reported there.

The number of rows per transmitted symbol, denoted as NPPS, is obtained from the number of captured image rows, the optical symbol period, and the camera frame period:

(6) $$\mathrm{NPPS}=N_{\mathrm{rows}}\frac{T_{\mathrm{sym}}}{T_{\mathrm{frame}}},$$

where $N_{\mathrm{rows}}$ is the number of captured image rows, $T_{\mathrm{sym}}$ is the optical symbol period, and $T_{\mathrm{frame}}$ is the camera frame period. The oscilloscope measurement gives an optical symbol rate of 3.7665kHz and therefore

(7) $$T_{\mathrm{sym}}=\frac{1}{3.7665\;\mathrm{kHz}}=265.50\;\mu s.$$

This OOK symbol rate is also the gross bit rate. With 32 transmitted symbols per character (including the two headers and packet repetition), the ideal unique-character rate is 3766.5/32=117.70 characters/s. The corresponding eight-bit payload rate is 941.6 bit/s, or 823.9 bit/s of seven-bit ASCII information, before camera gaps, missed packets, and higher-layer overhead. These are nominal protocol rates, not measured recovered goodput.

For the implemented system, $N_{\mathrm{rows}}=480$ and $T_{\mathrm{frame}}=9.678\;\mathrm{ms}$. Therefore,

(8) $$\mathrm{NPPS}=480\frac{265.50\;\mu s}{9.678\;\mathrm{ms}}=13.17\;\mathrm{rows}\;\mathrm{per}\;\mathrm{symbol}.$$

The value in Equation (8) is a nominal average derived from the configured frame period, not an independent measurement of active sensor-row timing; blanking and application delivery can affect the relationship. The CNN uses a fixed 105-row learned window, specified in Section 4. Separately, R=14 denotes the row-cell length used by the threshold reference procedure and the nominal synthetic symbol configuration.

A second dimensionless parameter characterizes how strongly the finite exposure interval degrades the received stripe pattern. Following Jurado-Verdu et al. [15], the exposure-to-symbol ratio is defined as the ratio between the exposure time and the optical symbol period,

(9) $$\mathrm{ESR}=\frac{T_{\exp}}{T_{\mathrm{sym}}},$$

where $T_{\exp}$ is the camera exposure time and $T_{\mathrm{sym}}$ is the symbol period given by Equation (7). Whereas NPPS describes the vertical sampling density, ESR quantifies the exposure-induced intersymbol interference introduced by the row integration of Equation (5): values above unity indicate that each row accumulates irradiance from more than one transmitted symbol. Using the effective exposure time of Table 1,

(10) $$\mathrm{ESR}=\frac{75\;\mu s}{265.50\;\mu s}=0.28.$$

This value is below the ESR≤0.5 regime identified by Jurado-Verdu et al. [15] as providing near-optimal sampling conditions, in which exposure-related intersymbol interference is negligible. This ratio is consistent with a short-exposure regime, but does not independently quantify the camera processing blur, row-time calibration, or all sources of intersymbol interference.

## 3. Datasets and Reference Labels

Both principal experiments use the same CNN input images and payload-output format, but differ in how their training labels are obtained.

**Synthetic-only training.** The CNN is trained only on synthetic images. Their appearance is calibrated using real development recordings, while their labels come directly from the packet positions and bits used to generate each image.
**Joint training on real recordings.** In a separate experiment, the CNN is trained on real images recorded at both 42 lx and 4 lx. Their payload labels are obtained using the fixed-threshold detector and the known transmitted sequence, with manual review of ambiguous cases.

Calibration, weight fitting, validation, and real evaluation are kept distinct below so that synthetic training is not confused with training on real target labels.

### 3.1. Real Recordings and ROI Representation

Several camera recordings were acquired while the transmitter repeatedly emitted predefined random, but known character sequences. The measured ROIs were converted into supervised examples using the offline procedure described in Section 3.2. The known sequence and the outputs of the classical receiver are used only to construct the target labels and are never supplied as CNN inputs.

Following this procedure, the supervised dataset is represented as

(11) $$D={\left\{\left(X_{m},Y_{m},v_{m}\right)\right\}}_{m=1}^{M},$$

with

(12) $$X_{m}\in {\left\{0,\ldots ,255\right\}}^{H\times W\times C},\;Y_{m}\in {\left\{0,1\right\}}^{S\times B},\;v_{m}\in {\left\{0,1\right\}}^{S},$$

where *M* is the number of retained ROI frames and each $X_{m}$ is the complete RGB ROI cropped around the LED footprint. The ROI dimensions are H=480 image rows, W=8 columns, and C=3 color channels. Here, B=8 is the number of MSB-first bits per character. As specified in Section 2.1, the transmitter sends every complete packet exactly twice to provide redundancy for the asynchronous camera receiver. Because packet transmission and camera-frame boundaries are not synchronized, one ROI can contain one or two complete payload observations. When two payloads are present, they may correspond either to repeated transmissions of the same character or to neighboring characters in the transmitted stream. The dataset therefore uses S=2 payload slots per frame. The target $Y_{m}$ stores the two possible payload bit vectors, while $v_{m}$ identifies which of the two slots contain a valid target. Bit values stored for an invalid slot are excluded from the bit loss.

Figure 5 shows this representation for a measured frame from the independent held-out capture. The annotated header and payload regions show how one complete payload observation is assigned to a valid slot. If another complete payload is visible in the same ROI, it is assigned to the second slot.

### 3.2. Reference Association and Manual Review

Real reference labels were constructed offline with a threshold receiver and alignment to a known cyclic transmitted string. They are reviewed proxy labels, not independently synchronized transmitter-log ground truth. For each ROI, the row profile is

(13) $$u_{m}\left[r\right]=\frac{1}{WC}\sum_{w=0}^{W-1}\sum_{d=1}^{C}X_{m}\left[r,w,d\right],\;r=0,\ldots ,479.$$

The original reference detector binarizes this profile using the strict decision $u_{m}\left[r\right]>2.0$ in native eight-bit intensity units. Bright runs of at least 76 rows define header candidates. The first row after the run is a payload start, provided that a complete eight-bit payload fits within the ROI. Each bit is decoded from 14 rows using a strict majority: more than seven bright rows decides one. These row-cell decisions belong to the offline reference detector, not the CNN’s 105-row learned window. At 4 lx, the reference procedure uses a recalibrated threshold of 1.0. The symbol support and header minimum remain unchanged. Effective thresholding in the original dark room does not imply that thresholding is a source of errors there.

Candidates are ordered chronologically and then vertically within each frame. Alignment searches possible initial phases of the known character cycle and accepts only an exact byte match within a bounded forward search of up to three expected characters. The preferred phase is selected by alignment coverage, candidate match rate, matched-candidate count, and fewer skips. Repeated observations and adjacent repeated characters require explicit handling; at most two detections are associated with one expected position. A matched expected index supplies the target byte and MSB-first bits. Crucially, before manual review, that expected byte is always equal to the detector’s decoded byte: a wrongly decoded byte is not automatically relabelled with the presumed transmitted character.

Unmatched candidates initially receive no supervised slot. A frame can remain if another candidate is aligned, in which case retained slots are compacted in vertical order. Frames with no aligned payload are excluded. An entirely missed payload generates no candidate and is not reconstructed from the known string or converted into an invented no-payload example. Skipped expected characters therefore do not constitute independently measured transmission losses.

Human review used the ROI, row-intensity profile, row decisions, candidate geometry, byte, sequence context, and neighboring frames. Fifteen previously unmatched physical candidates in Sources 10–15 were confirmed: twelve were second observations of an already associated character; three recovered the omitted sequence positions h@4948, z@4949, and w@4950. These three are part of the fifteen, not additional cases. Thirteen added slots belong to development and two to the holdout. Two previously excluded development frames were retained, producing 30,000 reviewed ROIs in total. This review resolved association/inclusion cases; it does not demonstrate correction of fifteen wrongly decoded bytes.

A complete, identifiable payload is retained even when the beginning of its header falls outside the ROI. Its payload labels remain valid, while the unknown header coordinates are marked as unavailable rather than assigned to row zero. These samples remain suitable for the bit and payload-validity training used in this study.

The verifier reconstructs each valid byte from the canonical expected stream and its stored index, and checks geometry, ordering, and source-level alignment gates. This strengthens auditability but is not independent verification of the physical temporal association. Because most labels were admitted through exact agreement with the fixed receiver, bit comparisons systematically favor that receiver. An unlabelled second slot is not always verified absence, and no transmitter-off reference set was collected.

### 3.3. Synthetic Generation and Calibration

Synthetic labels instead originate from packet positions and bytes sampled before image rendering; no threshold demodulator is used to label these samples. The renderer follows the finite-exposure observation model of Equation (5), using a continuous box-exposure integral and empirical ON/OFF appearance, spatial variation, and noise calibrated from Sources 10–14 [14,15]. This is a hybrid implementation, not a reproduction of a complete raw-sensor model.

Real captures thus provide appearance statistics for calibration, whereas the renderer supplies the labelled examples used for CNN weight fitting. This separation is the basis of the synthetic-only experiment: it avoids requiring real target-condition bit labels for optimization, but does not eliminate real measurements from the development workflow. All generated labels are simulator ground truth; their correctness by construction does not make the generated images a complete physical replica of the optical link.

For the 30 lx training experiment, calibration uses only the 42 lx development recordings. After rendering, processed channel intensities, including background and noise, are multiplied by the original drive-control scaling factor (approximately 0.7326) and rounded to eight-bit integers. This factor was used to generate the reported dataset; it is not the ratio of the subsequently reported illuminances, 30/42. This is an explicitly assumed proportional transformation, not a measured nonlinear optical transfer function. Scaling background and noise together is not an independent physical model of those components. The assumed exposure is one output-row period, the header support is 113 rows, and the symbol support is 14 rows. Independent random seeds produce 50,000 training, 5000 validation, and 5000 test frames; exact-frame hashes are checked for overlap within and between splits.

### 3.4. Dataset Partitions and Evaluation Scope

Table 2 lists the revised real-data partitions. No real test recording is used for weight fitting, validation, epoch selection, or batch-normalization updates in its corresponding experiment. However, these recordings have been inspected during the revision, so the analyses are retrospective capture-level tests, not a newly collected blind prospective validation. The original pre-review 29,998-frame dataset and its historical result are retained only for provenance and are not mixed with the reviewed results.

The principal experiments assign these recordings different roles. Synthetic-only training calibrates appearance using Sources 10–14 at 42 lx, fits and selects weights using generated data, and evaluates the frozen checkpoint on real 30 lx images. Joint-condition training fits one checkpoint using Sources 10–14 and 401–404 together, then evaluates it separately on Sources 15 and 405. The real 30 lx set is not a reported test of that joint checkpoint. This distinction prevents the two training demonstrations from being interpreted as one universal model.

The 23 excluded low-level development frames lack an accepted association. Two unmatched physical candidates remain outside the valid-slot reference in the low-level holdout. At 30 lx, 6489 of 6491 detected candidates were aligned. Accordingly, retention of all frames in either test set does not imply supervision of every visible payload. No dataset includes a verified all-empty scene, and reviewed datasets are not independent evidence of missed packets beyond the association process.

## 4. Receiver Architectures and Evaluation Protocol

The proposed receiver learns a mapping from a complete ROI to two ordered payloads and their validity. Its inference interface is unchanged between the synthetic-only and joint-condition experiments: only RGB pixels are supplied, without an illuminance identifier, an intensity threshold, or reference payload coordinates. This section specifies the 105-row architecture, followed by synthetic-only training and joint training on the two LED illuminance datasets. Supporting training experiments, comparison receivers, and scoring rules follow.

### 4.1. Static Phase Bank Architecture

The Static Phase Bank receives the complete 480×8 RGB ROI and applies a shared 105×8 learned window at every valid vertical position. For an ROI of height H=480 and a window length L=105, the candidate start positions are

(14) p∈P={0,1,…,H−L}={0,1,…,375},

These 376 positions form a fixed hypothesis axis that is preserved through the convolutional stages. The 105-row window is fixed for both principal training experiments.

Before entering the CNN, the ROI tensor $X_{m}$ defined in Section 3.1 is scaled from [0,255] to [0,1] and reordered to channel-first form, giving ${\tilde{X}}_{m}\in {\left[0,1\right]}^{C\times H\times W}$. No resizing or interpolation is applied. In Table 3, *k*, *s*, and *p* denote kernel size, stride, and zero padding, respectively.

The Static Phase Bank evaluates every start in Equation (14) using 24 shared kernels that span the three RGB channels and the complete 105×8 candidate window. Each kernel produces one scalar response at each start, resulting in a 24×376 feature map. The 24 channels are learned visual features, not character classes. Figure 6 summarizes the complete stage sequence, from the input ROI to the 18 receiver logits, together with the kernel geometry and tensor shape produced at each stage.

After the window bank, the network compares responses at neighboring starts and derives two latent slot-position score sequences. These internal scores are not independently supervised or used to select windows dynamically. They are fused with the learned features across adjacent positions, after which two fully connected layers produce the demodulator output. Table 3 gives the complete layer configuration and tensor dimensions.

The final 18-logit output is represented as

(15) $$f_{\theta }\left({\tilde{X}}_{m}\right)=\left({\hat{Z}}_{m},{\hat{v}}_{m}\right),\;{\hat{Z}}_{m}\in R^{2\times 8},\;{\hat{v}}_{m}\in R^{2}.$$

The first eight output values are the MSB-first bit logits for slot 1, the next eight are the corresponding logits for slot 2, and the final two are the slot-validity logits. The graph has 3,149,988 trainable parameters. All 376 hypotheses remain in the static graph: inference uses no dynamic crop, candidate routing, argmax, top-k, recurrent stage, or second model.

Synthetic-only fitting and joint fitting use this same 105-row architecture but produce separate checkpoints. An earlier CNN configuration, with 369 candidate starts and 3,096,676 parameters, is retained only for the supporting real-data comparisons; its geometry and results are identified separately.

### 4.2. Training and Adaptation Protocols

The bit loss excludes invalid reference slots, whereas both slot-validity outputs are supervised:

(16) $$\ell_{\mathrm{bit},m}=\frac{\sum_{j=1}^{2}\sum_{k=1}^{8}v_{m,j}\mathrm{BCE}\left({\hat{Z}}_{m,j,k},Y_{m,j,k}\right)}{8\sum_{j=1}^{2}v_{m,j}},\;\ell_{\mathrm{valid},m}=\frac{1}{2}\sum_{j=1}^{2}\mathrm{BCE}\left({\hat{v}}_{m,j},v_{m,j}\right),$$

(17) $$\ell_{m}=\ell_{\mathrm{bit},m}+0.25\ell_{\mathrm{valid},m}.$$

All retained training frames contain a valid slot. Optimization uses AdamW, learning rate $3\times 10^{-4}$, weight decay $10^{-4}$, batch size 256, FP32, dropout 0.10, and a maximum of 80 epochs. Real-data fitting uses no image augmentation and patience 20. Source-balanced loss is used for the reviewed original-model experiment; joint-condition fitting averages within each represented intensity group and then across groups. The two principal training routes below share the 105-row architecture but produce independent checkpoints.

#### 4.2.1. Synthetic-Only Fitting and Frozen Real Evaluation

The synthetic-only experiment uses the 50,000 generated training frames and the independent 5000-frame synthetic validation split from Section 3.3. Fitting uses seed 1234, the AdamW settings above, and patience 12. The best checkpoint, selected at epoch 55 using only synthetic validation, is evaluated on the distinct synthetic test set and then frozen for real 30 lx inference. It is not refitted on training plus validation. No real target frames are used for gradient updates, checkpoint selection, or batch-normalization adaptation in this run. Calibration from real 42 lx development recordings remains part of the workflow, and prior exploratory inspection prevents a claim of historically blind target-domain discovery.

#### 4.2.2. Joint High- and Low-Illuminance Fitting

Joint training starts from random initialization with seed 1234 and combines the normal- and low-level development recordings. Contiguous 250-frame blocks and source-specific split seeds 3456+source produce 36,000 inner-training and 8977 validation frames. Validation selects 44 epochs before reinitialization and full-development refitting on 44,977 frames. Sources 15 and 405 are excluded from fitting and selection. The resulting single checkpoint is frozen and tested independently at both illuminance levels. Group membership is used only to balance the training loss: inference supplies no illuminance value or condition identifier, and both tests use the same RGB scaling, weights, and zero-logit output decisions.

#### 4.2.3. Supporting Real-Data Training Experiments

The low-level 105-row control is trained from scratch with seed 1234 using Sources 401–404 alone. The block split provides 16,000 inner-training and 3977 validation frames; validation selects 25 epochs before full-development refitting. This control asks whether the joint checkpoint retains useful low-level performance compared with condition-specific fitting. It is not frozen low-level transfer from the original model.

For the earlier CNN configuration retained in Section 5.3, an all-development split uses seed 3456, with 20,000 training and 5000 validation frames, to select 70 epochs. The model is then reinitialized with seed 1234 and refitted on all 25,000 development frames before frozen Source-15 evaluation. This supporting comparison characterizes the earlier architecture/training configuration and is not substituted for either principal 105-row experiment.

### 4.3. Conventional and Representative CNN Baselines

All receiver comparisons use raw 480×8×3 ROIs without transmitted strings, expected indices, or reference payload coordinates as inference inputs. The fixed receiver uses Equation (13), thresholds 2.0 and 1.0 for the 42 lx and 4 lx recordings, respectively, a 76-row header minimum, 14-row symbol cells, and strict majority decisions. Candidates with complete payloads are ordered vertically.

The implemented automatic-threshold comparison replaces the fixed level by a per-frame Otsu threshold [8]:

(18) $$\tau_{m}=\arg\max_{\tau }\omega_{0}\left(\tau \right)\omega_{1}\left(\tau \right){\left[\mu_{0}\left(\tau \right)-\mu_{1}\left(\tau \right)\right]}^{2}.$$

Here $\omega_{i}$ and $\mu_{i}$ are the mass and mean of the two histogram classes. A 256-bin histogram spans the native floating-point range of the row means; the first maximizing split boundary is used. Constant profiles produce no candidates. Header and bit rules are otherwise identical to the fixed receiver. This is automatic *global* thresholding adapted between frames, not a spatially local adaptive-threshold algorithm. Its performance cannot stand for all adaptive-threshold approaches.

A spatially local comparison uses a one-dimensional adaptation of Bradley–Roth local-mean thresholding [23]. For the row profile of Equation (13), its decision level varies along the ROI:

(19) $$\begin{array}{c} \tau_{m}\left[r\right]=\frac{\alpha }{|N_{L_{a}}\left(r\right)|}\sum_{q\in N_{L_{a}}\left(r\right)}u_{m}\left[q\right],\\ N_{L_{a}}\left(r\right)=\left\{q\in \left\{0,\ldots ,479\right\}:|q-r\right|\leq \left(L_{a}-1\right)/2\}. \end{array}$$

Here $L_{a}$ is an odd averaging-window length in rows, distinct from the CNN’s learned window, and α is a dimensionless mean factor. Prefix sums compute centered local means; windows are clipped at ROI boundaries and divided by the number of observed rows, without padding. The strict decision $u_{m}\left[r\right]>\tau_{m}\left[r\right]$, 76-row header minimum, 14-row bit cells, complete-payload requirement, and strict majority decisions remain unchanged. Constant profiles produce no candidates. This is a row-profile OCC adaptation, not a reproduction of the original two-dimensional image experiment.

The predeclared 24-entry grid uses averaging-window lengths of 161, 225, 321, and 479 rows and mean factors of 0.20, 0.35, 0.50, 0.65, 0.85, and 1.00. All windows exceed the observed-header minimum. The same 250-frame development blocks and seeds 3456+source provide 5000 validation frames at 42 lx and 3977 at 4 lx. One shared setting is selected by maximizing the equally weighted mean of the two conditions’ exact-frame accuracies. Ties prefer greater coverage, lower conditional BER, and fewer validity false positives plus overflow candidates, followed by the first declared grid entry; an undefined conditional BER is ranked worst. The selected values, $L_{a}=321$ and α=0.35, are frozen before either test dataset is opened. Both test recordings use this setting without an illuminance input or condition-dependent switch.

The fixed-threshold and Otsu evaluations are complete for all 5000 test frames at each of 42 lx and 4 lx. A fresh classical-only run reproduced the archived framewise predictions and demodulation metrics, with dataset hashes checked and every prediction replayed from its raw ROI. This classical verification is separate from the neural comparison described below.

The local adaptive receiver is also evaluated on all 5000 test frames at each level, with the same reference arrays and masks as the fixed/Otsu runs. Independent convolution-based threshold computation and a separate binary-run/bit decoder reproduce its predictions on all 10,000 test frames and its selected-setting metrics on all 8977 validation frames. Dataset hashes, disjoint development/test images, frozen selection, and 12 receiver tests are checked; the 24 existing comparison regression tests also pass.

A representative non-phase-bank CNN uses a single InceptionTime-inspired backbone [24]. The RGB columns are losslessly rearranged into 24 channels over 480 rows. Six modules combine convolution branches with kernel lengths 40, 20, and 10, 32 filters per branch, and a pooled projection branch; residual connections follow modules three and six. An ordered head pools to 60 bins, flattens the representation, and maps it through 256 hidden units to the same 18 logits. It is an OCC adaptation, not the original InceptionTime ensemble.

The completed comparison matches this baseline to the jointly trained 105-row receiver using seed 1234. Both use the same pinned development datasets, 36,000/8977 inner-training/validation indices, epoch sample order, grouped loss, AdamW settings, 80-epoch limit, patience 20, and output decisions. Both consume the complete ROI scaled by 1/255, without an illuminance identifier or reference coordinates. Each architecture independently selects the lowest mean of the two validation reference-based BERs; ties prefer the lower worst-condition rate, fewer total bit errors, and the earlier epoch. The Inception adaptation selects 61 epochs, compared with 44 for the phase bank, and is reinitialized before refitting all 44,977 development frames. Its saved final checkpoint is then strictly reloaded and frozen before evaluating the two test recordings. This experiment does not evaluate a synthetic-only Inception model.

An independent verifier reloads both frozen networks and replays every test ROI, checking saved logits and decisions, reference arrays and masks, unchanged batch-normalization statistics, dataset/code/checkpoint hashes, validation selection, and matching development splits and epoch sample orders. It separately recomputes reference-based and conditional BER metrics and reproduces the phase bank’s original test metrics without changing that checkpoint. Six additional protocol/recovery tests and the 24 existing comparison tests pass.

### 4.4. Bit Error Rates and Statistical Reporting

Each output logit is decided at zero: z≥0 yields one. Eight MSB-first decisions form a byte; the two validity decisions determine which ordered slots are emitted. The bit error rate (BER) is the fraction of evaluated bits that differ from their reference values:

(20) $$\mathrm{BER}=\frac{\mathrm{Number}\;\mathrm{of}\;\mathrm{incorrect}\;\mathrm{bit}\;\mathrm{decisions}}{\mathrm{Total}\;\mathrm{number}\;\mathrm{of}\;\mathrm{evaluated}\;\mathrm{bits}}.$$

Correct bits divided by evaluated bits is *bit accuracy*, equal to 1−BER, rather than BER.

The tables identify which bits are evaluated. For CNN bit-prediction results, ${\mathrm{BER}}_{\mathrm{ref}}$ includes all reference-labelled payload bits, irrespective of the CNN’s validity decision. For receiver comparisons, ${\mathrm{BER}}_{\det}$ includes only slots marked valid by both the reference and the receiver, because a missed conventional detection has no decoded bits. Both use Equation (20), but their evaluated-bit totals can differ.

To report missed payloads separately, conditional BER is accompanied by reference-slot coverage:

(21) $$C=\frac{\mathrm{Reference}\text{-}\mathrm{valid}\;\mathrm{slots}\;\mathrm{marked}\;\mathrm{valid}\;\mathrm{by}\;\mathrm{the}\;\mathrm{receiver}}{\mathrm{Total}\;\mathrm{reference}\text{-}\mathrm{valid}\;\mathrm{slots}}.$$

A low conditional BER does not imply high coverage. If no reference-valid payload is detected, coverage is zero and conditional BER is undefined, not zero. Missed payloads are not assigned invented bit values. Ordering is vertical, not geometry-matched, so an extra or missed early candidate can shift subsequent slot comparisons.

Byte agreement requires all eight bits to match on a reference-valid slot; correctly emitted bytes additionally require predicted validity. Validity precision, recall, and F1 use true-positive, false-positive, and false-negative reference slot counts. Their false positives are not necessarily physically absent payloads, because the labels are incomplete proxies. Exact-frame recovery requires correct validity and all valid bytes, and in the classical comparison also requires no candidate overflow beyond two slots.

Counts and denominators accompany rates. The principal CNN experiments and matched neural comparison each use one training seed, so they do not quantify variation across training seeds. No independent-bit binomial interval or statistical superiority claim is inferred from correlated repeated packets.

Real-data BER is measured against reviewed proxy labels, whereas synthetic-data BER uses simulator-known bits. The former is not an independently measured transmitted-stream BER. Missed transmissions, repetition combining, stream ordering, and empty-scene false alarms require additional reference instrumentation.

## 5. Results and Discussion

The results first address synthetic-to-real demodulation and then joint training for operation at both LED intensity levels with one checkpoint. Conventional and matched single-seed CNN comparisons establish the context and limits of those findings. Unless simulator ground truth is explicitly identified, agreement is measured against real-image proxy labels, not independently synchronized physical stream bits.

### 5.1. Synthetic-Only Training and Real-Image Demodulation

Table 4 evaluates the same synthetic-only 105-row checkpoint on generated and real images. No real images or real bit labels enter CNN weight fitting or checkpoint selection in this experiment; the real development captures supply only the generator’s appearance calibration. The real target-level evaluation is performed frozen, without fine-tuning.

On real images, the network gives 93 disagreements in 51,912 reference bits and recovers all labelled payloads and slot-validity decisions in 97.6% of frames. It matches 6422/6489 reference bytes, of which 6400 are also emitted as valid. Reference-slot validity counts are TP = 6467, FP = 41, and FN = 22. All 93 bit disagreements occur in slot zero; this observation alone does not establish their physical cause. Performance is lower than on the synthetic test set, but remains useful for the demonstrated frame-local demodulation task.

This is the first principal finding: calibrated synthetic examples can supply the CNN’s entire labelled fitting set while retaining useful performance on real images at the target illuminance level. The result goes beyond accuracy on generated data because the same checkpoint, selected at epoch 55 using synthetic validation only, is applied to measured camera observations. It does not demonstrate calibration-free transfer, a quantified reduction in laboratory effort, or replacement of all real measurements. Appearance calibration used five 42 lx development recordings, not a demonstrated single-capture experiment. Synthetic data could also be used to tune conventional receivers; that alternative has not been compared here.

### 5.2. One Jointly Trained Network for Two LED Intensity Conditions

Table 5 tests a separate 105-row checkpoint trained jointly on 42 lx and 4 lx development recordings. The two joint-training rows use exactly the same weights and input preprocessing; no condition identifier, model switch, or external threshold adjustment is supplied at inference. The low-level scratch model is a condition-specific training control, not that joint checkpoint.

The single joint checkpoint gives 12/51,968 bit disagreements at 42 lx and 21/51,696 at 4 lx, with exact-frame recovery of 99.64% and 99.50%, respectively. Thus, one fitted receiver can incorporate both represented conditions and demodulate separate recordings at each without changing its parameters. At 4 lx, the joint and condition-specific models give 21 and 18 disagreements, respectively. These single-seed counts support useful operation under joint training, not statistical equivalence or superiority between fitting strategies.

This is the second principal finding: the two measured LED intensity conditions can be incorporated into one learned receiver rather than requiring condition-dependent model selection. The completed fixed-threshold comparisons instead use separately selected levels of 2.0 and 1.0. The local adaptive receiver in Section 5.3 also operates at both levels using one shared parameter setting. Multi-condition operation is therefore a demonstrated CNN capability, not an exclusive advantage over conventional reception. These results concern the two reported receiver-plane illuminances and do not establish robustness to arbitrary illumination or unrepresented conditions.

These are joint-training results, not frozen transfer of the earlier CNN checkpoint. Comparing them with the original Source-15 result also changes window geometry and training distribution, so the improvement cannot be attributed solely to illumination coverage. The synthetic-only checkpoint in Section 5.1 is a different model; the two demonstrations must not be combined into a claim that one tested checkpoint covers every reported illuminance level.

### 5.3. Receiver Comparisons

The training capabilities above should be distinguished from a claim of better demodulation accuracy than conventional receivers. Table 6 reports completed conventional evaluations and a matched joint-training CNN comparison on the revised normal and low-level test ROIs. The two joint CNNs use the seed-1234 protocol in Section 4.3. The earlier CNN configuration’s Source-15 predictions were additionally rescored from their archived per-frame bit and validity outputs using conditional BER and coverage as defined in Section 4.4. This rescoring does not retrain the model. The earlier configuration remains a separate supporting result, not one of the two matched jointly trained networks.

The fixed receiver’s exact agreement with valid reference bits is consistent with label provenance; it is not independent evidence of zero physical BER. Its two extra low-level detections occur outside the proxy-valid slots. No completed test reports candidate overflow in this table.

At 42 lx, Otsu gives 39 disagreements in 50,536 jointly valid bits, with 97.24% reference-slot coverage and 96.42% exact-frame recovery. At 4 lx, all 5000 frame evaluations completed, but none produced an accepted payload candidate. This is a completed decoding failure, not a missing or interrupted evaluation.

The 4 lx Otsu thresholds range from 2.8875 to 3.7100 in native image-intensity units, with a median of 3.3008. After thresholding, the longest consecutive bright run in each frame spans only 14–20 rows, below the unchanged 76-row header minimum. No header therefore qualifies, and no bits enter the conditional comparison; conditional BER is undefined, not zero. This failure belongs to this specific histogram/packet-decoder configuration; it does not demonstrate that adaptive thresholding is inherently unsuitable.

With the same frozen $L_{a}=321$, α=0.35 setting at both levels, the local-mean receiver gives 18/51,848 conditional bit disagreements at 42 lx and 4/51,672 at 4 lx. Its reference-slot coverage is 99.77% and 99.95%, and exact-frame recovery is 99.26% and 99.76%, respectively. It misses 15 and 3 reference slots and produces 22 and 6 validity false positives relative to the proxy labels. These false positives are not a verified empty-scene false-alarm count. Thresholds vary along the ROIs and between frames, spanning 6.1745–11.1815 native intensity units at 42 lx and 0.6462–1.3329 at 4 lx.

This local adaptation improves coverage and exact-frame recovery over the implemented global Otsu receiver at both levels, but does not outperform the condition-specific fixed receiver on these proxy-labelled recordings. It also demonstrates conventional operation across both measured conditions without a supplied illuminance or externally switched setting. Consequently, the CNN’s joint-training result should be interpreted as useful learned multi-condition operation, not evidence that only a CNN can provide it. The two bit metrics used in Table 5 and Table 6 have different validity denominators and must not be ranked as if identical.

### 5.4. Computational Cost and Deployment Feasibility

For one ROI, convolution and dense-layer multiply–accumulate (MAC) counts are obtained directly from tensor geometry. Table 7 excludes batch normalization, activation functions, memory movement, and framework overhead; one MAC is not counted as one FLOP.

For the evaluated 105-row CNN, the first dense layer contains 12,032 × 256 weights and 256 biases, approximately 97.8% of the parameters. Conversely, the large window bank dominates arithmetic. The eight-bit ROI occupies 11,520 bytes; its FP32 form occupies 46,080 bytes. The largest exposed feature map is 32×376 values, but this is not peak runtime memory. Simultaneously live tensors, concatenations, convolution workspaces, parameter buffers, allocator behavior, and quantization metadata must also be counted.

An archived batch-one CPU probe of the original model, using PyTorch (version 2.8.0) with one thread, 20 warmups, and 100 model-only calls on a zero tensor, records a mean of 1.045 ms and a 95th percentile of 1.053 ms on the GPU workstation (Intel Core i7-14700). It excludes ROI acquisition and preprocessing and is not an embedded-device latency measurement. In a separate receiver probe including row-profile extraction, the fixed receiver’s medians are approximately 0.039 ms at both illuminance levels, versus 0.094 ms and 0.089 ms for Otsu. These probes have different input and preprocessing scopes, so they do not constitute an end-to-end speedup comparison.

The local-mean receiver has no learned parameters and uses a linear-time prefix-sum computation over the row profile. A separate batch-one CPU probe gives approximately 0.055 ms median latency at both levels, including raw-ROI row-mean extraction, local threshold computation, and packet decoding, but excluding disk I/O. It uses 20 warmups and 100 calls; this additional probe is not a simultaneous end-to-end timing comparison with the archived receivers or an embedded-device measurement.

The matched Inception adaptation contains 2,465,042 parameters, requires 238,146,048 convolution/dense MACs per ROI, and occupies 9.860 MB of FP32 parameter storage. Inception therefore requires approximately 8.68 times the phase bank’s counted MACs, whereas the phase bank uses more parameters and storage (3,149,988 and 12.600 MB). Lower arithmetic cost does not imply a smaller parameter footprint or measured peak memory.

A matched batch-one workstation probe uses one PyTorch CPU thread, 20 warmups, and 100 calls per model and condition. Including raw-ROI conversion, 1/255 scaling, and inference but excluding disk I/O, CPU median latencies at 42/4 lx are 1.033/1.036 ms for the phase bank and 4.345/4.340 ms for Inception. A separate synchronized model-only probe on resident tensors in an NVIDIA RTX 3060 gives GPU medians of 0.178/0.178 ms and 1.204/1.201 ms, respectively. These new measurements compare the two CNNs under matched probe scopes; they do not replace the historical timings above or include image acquisition, disk I/O, or embedded execution.

The conventional receiver has no learned parameters and is substantially simpler than either CNN. The phase bank’s lower MAC count and workstation latency relative to this adapted backbone do not establish general compactness across task-matched CNNs; measured peak memory remains unavailable. A fixed-shape graph is only a potential pathway to edge-camera deployment. No IMX500 conversion, supported-layer certification, quantization-accuracy result, power measurement, or on-device runtime is claimed; apparent parameter-storage fit alone would not establish deployment feasibility.

### 5.5. Limitations and Future Directions

The demonstrated contributions are limited to the calibrated synthetic-training route and the two represented real illumination conditions. Neither experiment establishes arbitrary-condition robustness, the minimum number of calibration captures, or an accuracy advantage over conventional reception. Synthetic-only and joint-condition fitting also use different checkpoints, and multi-seed uncertainty is unavailable for these principal experiments.

The principal reference limitation is selection through threshold decoding and exact sequence matching. Human review improves completeness, but does not turn these labels into physical ground truth or measure all missed transmissions. Future captures should include independent transmitter packet identifiers or timestamps, explicit transmitter-off scenes, and reviewed threshold-failure payloads. Receiver comparisons should be repeated on that independently associated corpus, including a conventional receiver calibrated with the same synthetic information and a shared-threshold test across the two illuminance levels.

A payload-local bit decoder, a separate synchronization/validity branch, and context-consistency training that perturbs pixels outside a known training payload are concrete directions to test, not demonstrated improvements. A lighter head could also reduce the dense parameter bottleneck. The completed input-matched Inception comparison should be repeated across seeds and recordings, alongside the requested ablations, to determine whether its observed differences persist and which architectural choices are useful.

The demonstrated training flexibility does not replace prospective cross-session validation. Future experiments should separately vary LED drive, exposure, ROI offsets, camera timing, distance, and angle, reporting frozen inference first and adaptation second. Synthetic domain randomization should include independently modeled background/noise, boundary-censored headers, multiple measured camera settings, and timing variation, with calibration sources separated from all target tests. A next experiment could combine synthetic-only fitting and multi-condition coverage in one checkpoint; that combined capability is not claimed from the current separate demonstrations.

Finally, a device-specific deployment study must measure conversion success, peak memory, INT8 accuracy, input-row preservation, latency, and power. The dark-room demonstrator is intended to represent the low-ambient-light aspect of intra-satellite operation, so illumination representativeness and full spacecraft qualification are distinct questions. Spacecraft application still requires additional optical integration, environmental qualification, fault handling, and link-level reliability analysis beyond this laboratory receiver study.

## 6. Conclusions

This work proposed and evaluated the Static Phase Bank CNN for demodulating LED-transmitted OOK signals from rolling-shutter camera images. The 40 cm, nominally face-to-face laboratory link operates at a gross rate of 3.77kbit/s and serves as a ground-based analogue of short-range intra-satellite communication, with dark-room acquisition representing the expected low-ambient-light condition inside the enclosed satellite. The receiver predicts two ordered payloads and their validity from the complete ROI without an external intensity threshold or payload-start detector.

The first principal result is synthetic-to-real demodulation. The 105-row CNN trained exclusively on generated frames, with appearance calibrated from real development recordings, gives 93 disagreements in 51,912 proxy-labelled real bits ($1.791\times 10^{-3}$) and 97.6% exact-frame recovery without target-level fine-tuning. Simulator labels supply the fitting targets; real captures remain necessary for appearance calibration. This demonstrates useful real-image performance after synthetic-only fitting, not a calibration-free receiver.

The second principal result is multi-condition operation with one network. A separate 105-row checkpoint trained jointly on high- and low-illuminance recordings gives 12/51,968 and 21/51,696 bit disagreements on the respective test recordings, with 99.64% and 99.50% exact-frame recovery. Both conditions use the same weights, preprocessing, and output rules, without condition-dependent model switching or an externally adjusted intensity threshold.

A local-mean adaptive conventional receiver likewise uses one development-selected parameter setting at both levels, recovering exact frames in 99.26% and 99.76% of cases. Thus, operation across these two conditions is not exclusive to neural reception; the CNN result demonstrates the capability within its learned image-to-payload mapping.

In a matched seed-1234 joint-training comparison, the phase bank recovers exact frames more often than the InceptionTime-inspired OCC adaptation at both levels (99.64/99.50% versus 98.14/96.68%). It also has lower convolution/dense MAC counts and measured workstation latency, but more parameters. This is an observed whole-network comparison, not proof of general architectural superiority or of the contribution of each phase-bank component.

These findings do not establish general robustness or an accuracy advantage over conventional receivers. Fixed reception agrees with the proxy-valid bits in the comparison recordings, consistent with threshold-based label selection. Independent transmitter references, multi-seed CNN comparisons, component ablations, broader acquisition sweeps, and actual device conversion remain necessary. Within this scope, the two practical contributions are training on synthetic frames while retaining useful real-image demodulation, and incorporating multiple measured illumination conditions into a single learned receiver.

## Figures and Tables

**Figure 1:**
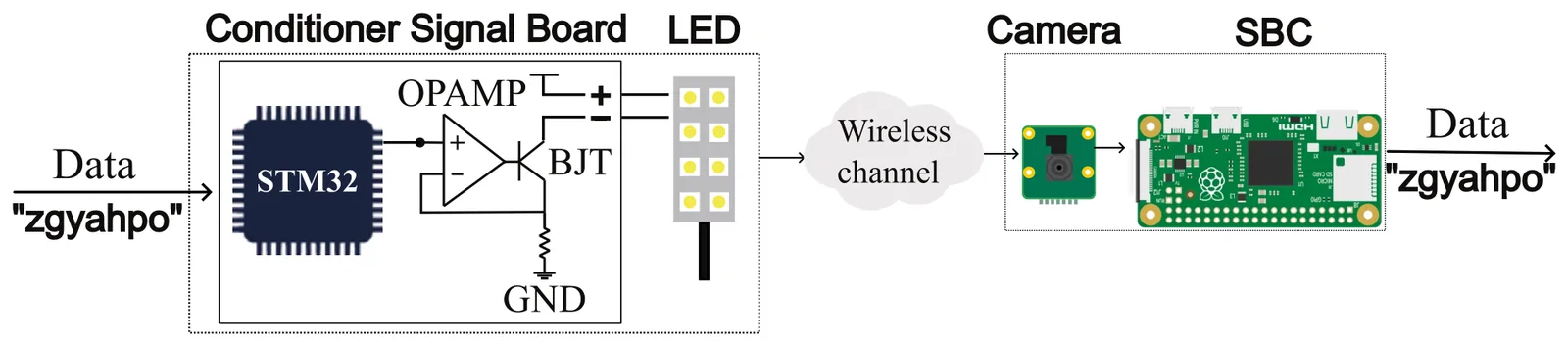
End-to-end laboratory RS-OCC chain. The conditioner and signal board encodes the input data and drives the LED transmitter. The emitted optical signal is captured by the camera receiver, after which the single-board computer performs ROI extraction and packet-compatible demodulation.

**Figure 2:**
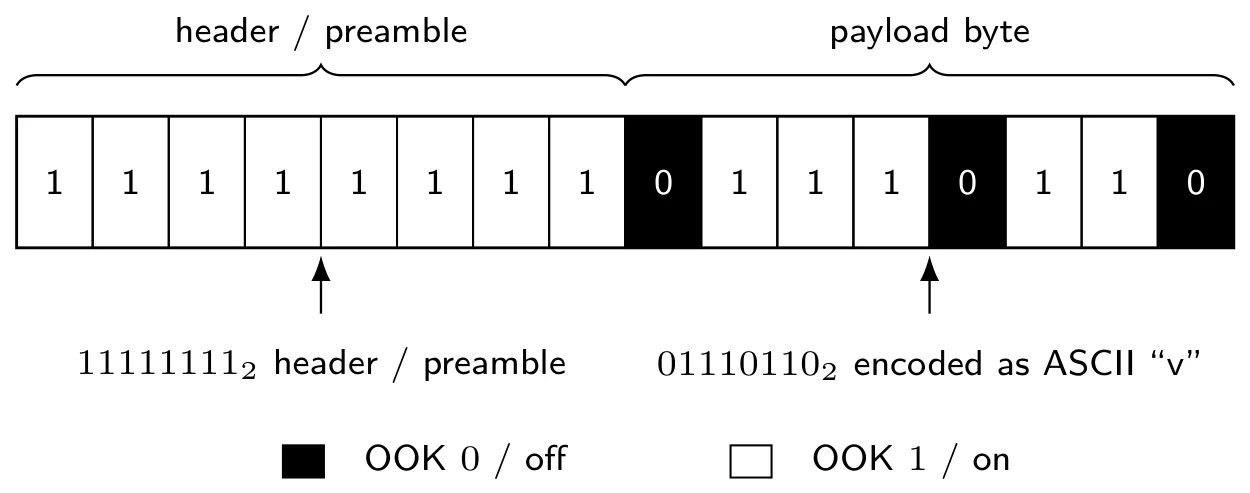
Simplified OOK packet structure used by the RS-OCC receiver. Black and white fields denote binary zero and one optical states, respectively. The illustrated packet contains an eight-bit header/preamble followed by one eight-bit ASCII payload byte.

**Figure 3:**
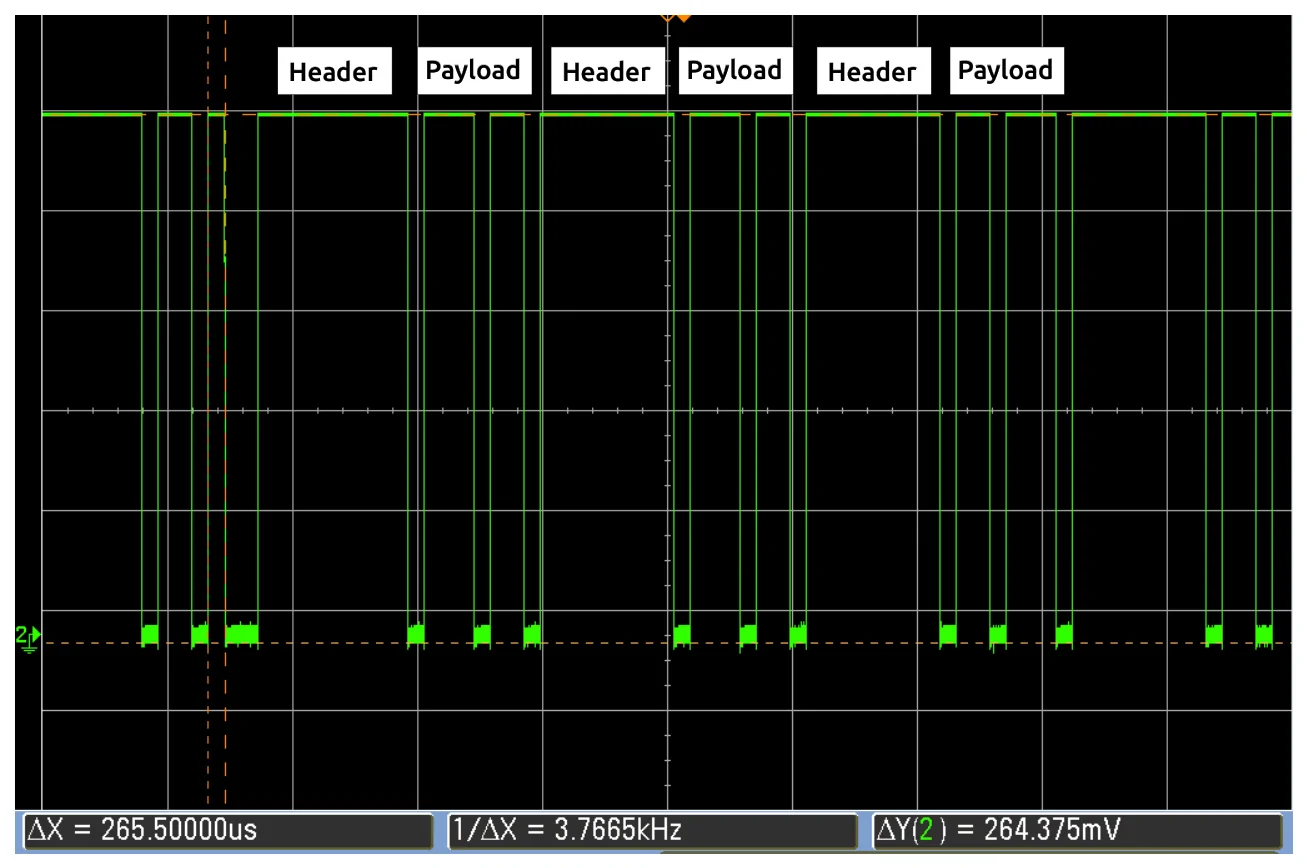
Representative oscilloscope trace of the transmitted OOK waveform. The green trace shows the LED drive signal, and the annotated regions identify the consecutive synchronization headers and ASCII payloads. The vertical orange dashed lines are time cursors placed at the start and end of one symbol; their separation, ΔX=265.50μs, is the symbol period, and 1/ΔX=3.7665 kHz is the symbol rate. The horizontal dashed lines are the amplitude cursors (ΔY). Packets at the left and right edges are truncated by the oscilloscope capture window.

**Figure 4:**
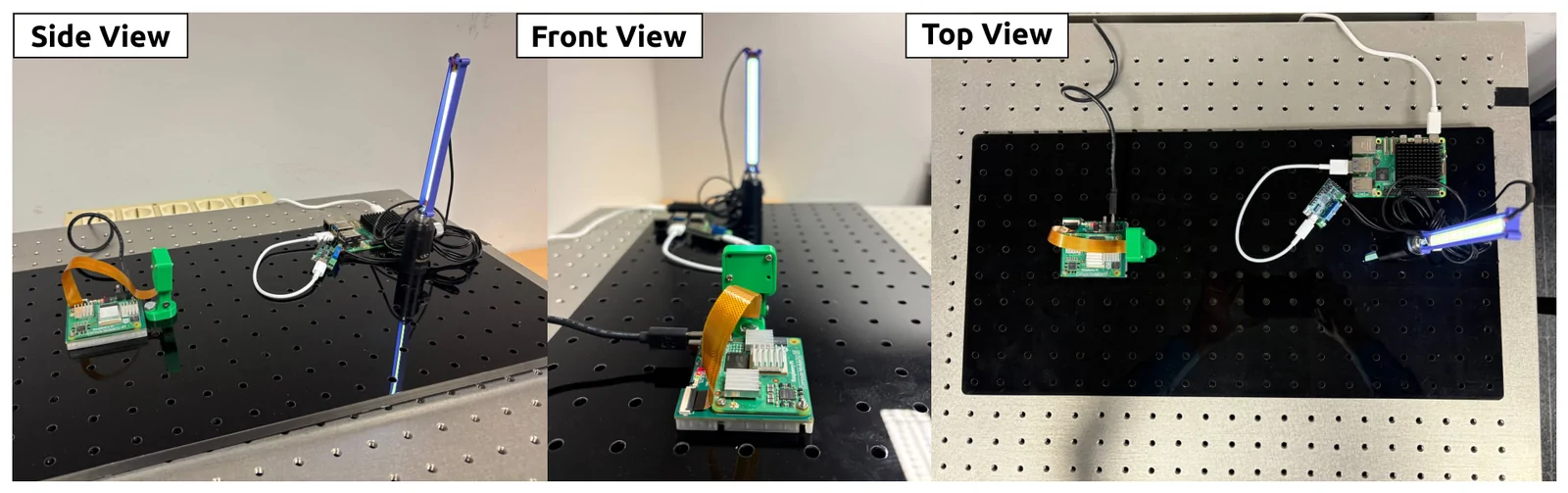
Experimental RS-OCC setup used to acquire the laboratory recordings. The modulated optical source, camera receiver, and embedded processing hardware are mounted on an optical breadboard; their relative geometry is controlled during acquisition. The RGB acquisition parameters are listed in Table 1.

**Figure 5:**
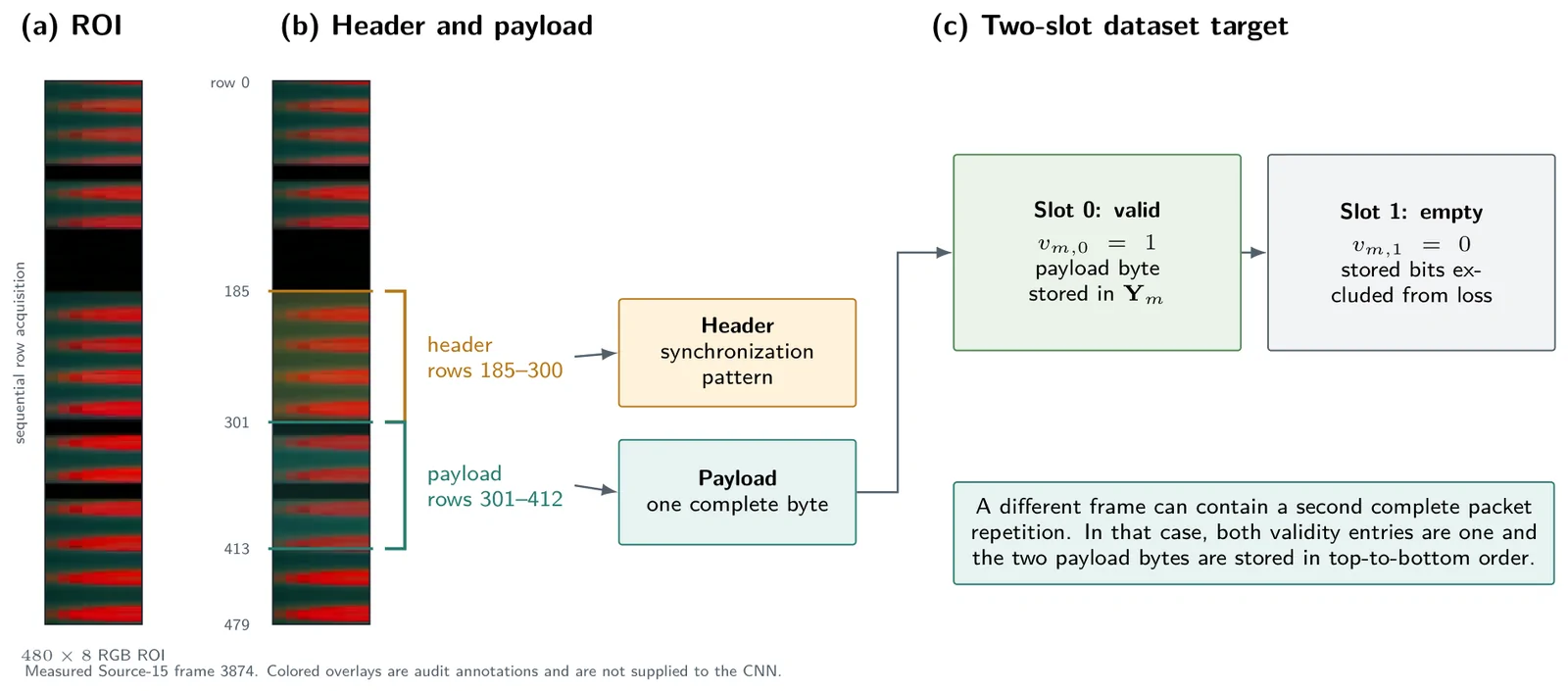
Measured rolling-shutter ROI and its two-slot dataset representation. For this example, the targets are $Y_{m}=\left[{\left(01110111\right)}_{2},{\left(00000000\right)}_{2}\right]$ and $v_{m}=\left[1,0\right]$. Thus, slot 0 contains one valid payload and slot 1 has no accepted reference payload; the placeholder zeros in the invalid slot are excluded from the bit loss. Header rows 185–300 and payload rows 301–412 are audit annotations used to explain label construction and are not CNN inputs.

**Figure 6:**
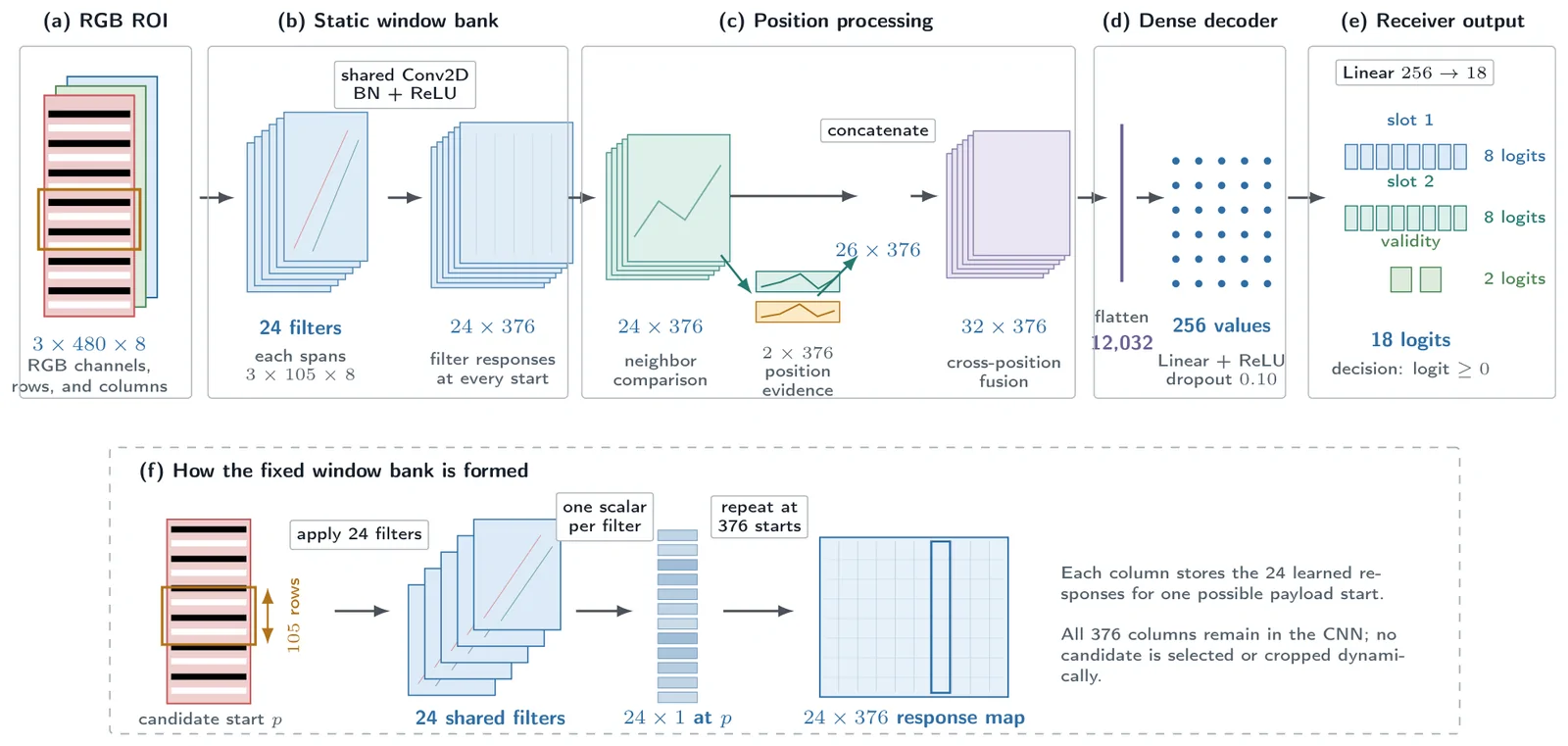
Static Phase Bank CNN architecture, shown stage by stage. Grey frames group the processing stages (**a**–**e**), and the dashed frame encloses the explanatory inset (**f**). Red, green, and blue sheets are the three color channels of the ROI, and the orange box marks one 105-row candidate window. Blue denotes window-bank filters and responses; teal and orange denote the neighbor-comparison features and the latent slot-position evidence; purple denotes the fused features. In (**e**), blue, teal, and green denote the slot-1 bit logits, slot-2 bit logits, and validity logits. Stacked sheets represent channels or filters. (**a**) The complete 3×480×8 RGB ROI is supplied to the network. (**b**) A bank of 24 shared 3×105×8 filters evaluates all 376 unit-stride starts, producing a 24×376 response map. (**c**) Neighbor comparison preserves that shape, two pointwise channels provide a 2×376 latent slot-position map, and concatenation followed by cross-position fusion produces 32×376 features. (**d**) Flattening yields 12,032 values, which feed a 256-unit hidden layer with ReLU and dropout 0.10. (**e**) The output contains eight MSB-first bit logits per slot and two validity logits; a logit greater than or equal to zero decides one. (**f**) The inset illustrates how each candidate start contributes one column of learned responses to the fixed bank. Tensor dimensions omit the batch axis and the singleton width after the first convolution.

**Table 1:** Laboratory acquisition parameters for the RGB ROI dataset.

Parameter	Value
Optical transmitter	LED strip behind diffuser
LED-to-camera distance	40 cm
Viewing geometry	Face-to-face, nominally normal incidence
Receiver-plane illuminance	42, 30, and 4 lx
Ambient illumination	Dark room, negligible ambient light
Receiver interface and format	Picamera2, RGB888
Resolution	640×480 pixels
Frame rate	103.33 fps
Effective exposure time	75μs
Analogue gain	1.0
Camera buffer count	8 frames
CNN ROI (height × width × channels)	480×8×3
Source captures	6 captures × 5000 frames each
Reviewed normal-level RGB ROI frames	30,000

**Table 2:** Real recordings and their use in the experiments. Generator calibration uses real images to set synthetic appearance, not to fit CNN weights in the synthetic-only experiment. Reference slots are proxy labels; valid bits equal eight times the valid slots.

Illuminance (lx)	Use in Experiments	Raw Frames	Retained Frames	Valid Slots	Valid Bits
42	Generator calibration; real-data training/validation	25,000	25,000	32,458	259,664
42	Real-data test	5000	5000	6496	51,968
4	Real-data training/validation	20,000	19,977	25,808	206,464
4	Real-data test	5000	5000	6462	51,696
30	Synthetic-to-real test	5000	5000	6489	51,912

**Table 3:** Layer-by-layer specification of the Static Phase Bank CNN. Tensor dimensions omit the training batch axis and the singleton width dimension after the first convolution.

Stage	Operation	Input	Configuration	Output
Window bank	Conv2D + BN + ReLU	3×480×8	3→24, k=105×8, s=1, p=0	24×376
Neighbor comparison	Conv1D + BN + ReLU	24×376	24→24, k=3, s=1, p=1	24×376
Slot-position evidence	Conv1D	24×376	24→2, k=1, s=1, p=0	2×376
Concatenation	Channel concatenation	24×376 and 2×376	No trainable parameters	26×376
Cross-position fusion	Conv1D + BN + ReLU	26×376	26→32, k=3, s=1, p=1	32×376
Flatten	Reshape	32×376	No trainable parameters	12,032
Hidden layer	Linear + ReLU + dropout	12,032	12,032→256, dropout 0.10	256
Output layer	Linear	256	256→18	18 logits

**Table 4:** Synthetic-only 105-row CNN tested on 5000 generated frames (simulator ground truth) and 5000 real 30 lx frames (proxy labels). Both rows use the same frozen checkpoint. ${\mathrm{BER}}_{\mathrm{ref}}$ scores all reference-valid bits; byte agreement does not require predicted validity.

Test Images	Errors/Reference Bits	${\mathrm{BER}}_{\mathrm{ref}}$	Byte Agreement	Validity F1	Exact Frames
Synthetic, 30 lx target	7/51,640	$1.356\times 10^{-4}$	99.9225%	99.9535%	4990/5000
Real, 30 lx	93/51,912	$1.791\times 10^{-3}$	98.9675%	99.5153%	4880/5000

**Table 5:** Real-data fitting experiments for the 105-row configuration, with a separate 5000-frame test recording at each illuminance level. Both joint rows use one checkpoint. ${\mathrm{BER}}_{\mathrm{ref}}$ scores all reference-valid bits; byte agreement does not require predicted validity.

Real Fitting Illuminance (lx)	Real Test Illuminance (lx)	Errors/ Reference Bits	${\mathrm{BER}}_{\mathrm{ref}}$	Byte Agreement	Validity F1	Exact Frames
4 only	4	18/51,696	$3.482\times 10^{-4}$	99.8298%	99.8375%	4971/5000
42 + 4	42	12/51,968	$2.309\times 10^{-4}$	99.8461%	99.9230%	4982/5000
42 + 4	4	21/51,696	$4.062\times 10^{-4}$	99.7679%	99.8839%	4975/5000

**Table 6:** Completed receiver comparisons on the same retained ROIs within each illuminance level. ${\mathrm{BER}}_{\det}$ is conditional BER; *C* is reference-slot coverage. Zero-error entries use <1/N as a one-error resolution convention, not a statistical upper bound; *N* is the number of evaluated bits. Undefined means no jointly valid bits. Exact recovery also penalizes extra candidates. Local mean uses one development-selected setting at both levels; each joint CNN uses one checkpoint at both levels, with matched seed-1234 fitting. The earlier CNN configuration is not part of that matched comparison.

Illuminance (lx)	Receiver	Errors/Evaluated Bits	${\mathrm{BER}}_{\det}$	*C*	Correctly Emitted Bytes	Exact Frames
42	Fixed, τ=2.0	0/51,968	<$1.924\times 10^{-5}$	100.00%	6496	5000/5000
42	Per-frame Otsu	39/50,536	$7.717\times 10^{-4}$	97.24%	6302	4821/5000
42	Local mean, shared setting	18/51,848	$3.472\times 10^{-4}$	99.77%	6474	4963/5000
42	Joint 105-row phase bank	12/51,912	$2.312\times 10^{-4}$	99.89%	6479	4982/5000
42	Joint Inception adaptation	93/51,960	$1.790\times 10^{-3}$	99.98%	6405	4907/5000
42	Earlier CNN configuration	1325/51,864	$2.555\times 10^{-2}$	99.80%	5241	3756/5000
4	Fixed, τ=1.0	0/51,696	<$1.934\times 10^{-5}$	100.00%	6462	4998/5000
4	Per-frame Otsu	0/0	Undefined	0%	0	0/5000
4	Local mean, shared setting	4/51,672	$7.741\times 10^{-5}$	99.95%	6455	4988/5000
4	Joint 105-row phase bank	21/51,632	$4.067\times 10^{-4}$	99.88%	6439	4975/5000
4	Joint Inception adaptation	160/51,648	$3.098\times 10^{-3}$	99.91%	6301	4834/5000

**Table 7:** Analytical neural-network resource accounting. Decimal MB are used. INT8 values are hypothetical one-byte-per-parameter storage, not converted model sizes.

Quantity	Earlier CNN	Evaluated 105 Rows
Candidate starts	369	376
Trainable parameters	3,096,676	3,149,988
Window-bank MACs	23,804,928	22,740,480
Other convolution MACs	1,576,368	1,606,272
Dense-layer MACs	3,027,456	3,084,800
Total MACs/ROI	28,408,752	27,431,552
FP32 parameter storage	12.387 MB	12.600 MB
Hypothetical INT8 parameter storage	3.097 MB	3.150 MB
Largest exposed feature map, FP32	47,232 bytes	48,128 bytes

## Data Availability

The data presented in this study are available on request from the corresponding author. The data are not publicly available because they were generated within the OCC4SAT project (European Union Horizon Europe research and innovation programme, Grant Agreement No. 101135434) and their dissemination is governed by the project consortium agreement, which does not permit public release before the end of the project. The datasets will be deposited in a public repository once the project is completed.

## Abbreviations

The following abbreviations are used in this manuscript:

AdamW	Adam optimizer with decoupled weight decay
ASCII	American Standard Code for Information Interchange
BER	Bit error rate
BN	Batch normalization
CNN	Convolutional neural network
ESR	Exposure-to-symbol ratio
ISI	Intersymbol interference
LED	Light-emitting diode
MIMO	Multiple-input multiple-output
MSB	Most significant bit
NPPS	Number of pixels per symbol
OCC	Optical Camera Communication
OFDM	Orthogonal frequency-division multiplexing
OOK	On–Off Keying
OWC	Optical wireless communication
ReLU	Rectified linear unit
RF	Radio frequency
RGB	Red, green, blue
ROI	Region of interest
RS	Rolling shutter
VLC	Visible light communication

## References

[1] Ferrari F., Rabadan J.A., Guerra V., Giuliani M., Nicolle S., Bataillou B., Perez-Jimenez R. OCC4SAT: Optical Camera Communications for intra-satellite data transfer. Proceedings of the 75th International Astronautical Congress (IAC 2024).

[2] Rabadan J.A., Guerra V., Ferrari F., Perez-Jimenez R., Giuliani M., Bataillou B., Nicolle S. (2024). OCC strategies for intra-satellite communications: OCC4SAT project. Proceedings of the 2024 14th International Symposium on Communication Systems, Networks and Digital Signal Processing (CSNDSP), Rome, Italy, 17–19 July 2024.

[3] Saha N., Ifthekhar M.S., Le N.T., Jang Y.M. (2015). Survey on optical camera communications: Challenges and opportunities. IET Optoelectron..

[4] Saeed N., Guo S., Park K.-H., Al-Naffouri T.Y., Alouini M.-S. (2019). Optical camera communications: Survey, use cases, challenges, and future trends. Phys. Commun..

[5] Nguyen T., Hossain M.A., Jang Y.M. (2016). Design and implementation of a novel compatible encoding scheme in the time domain for image sensor communication. Sensors.

[6] (2019). IEEE Standard for Local and Metropolitan Area Networks–Part 15.7: Short-Range Optical Wireless Communications.

[7] Liang K., Chow C.-W., Liu Y., Yeh C.-H. (2016). Thresholding schemes for visible light communications with CMOS camera using entropy-based algorithms. Opt. Express.

[8] Otsu N. (1979). A threshold selection method from gray-level histograms. IEEE Trans. Syst. Man Cybern..

[9] O’Shea T.J., Hoydis J. (2017). An introduction to deep learning for the physical layer. IEEE Trans. Cogn. Commun. Netw..

[10] Lee H., Lee S.H., Quek T.Q.S., Lee I. (2019). Deep learning framework for wireless systems: Applications to optical wireless communications. IEEE Commun. Mag..

[11] Ma S., Dai J., Lu S., Li H., Zhang H., Du C., Li S. (2019). Signal demodulation with machine learning methods for physical layer visible light communications: Prototype platform, open dataset, and algorithms. IEEE Access.

[12] Liu L., Deng R., Chen L.-K. (2019). 47-kbit/s RGB-LED-based optical camera communication based on 2D-CNN and XOR-based data loss compensation. Opt. Express.

[13] Lin Y.-S., Chow C.-W., Liu Y., Chang Y.-H., Lin K.-H., Wang Y.-C., Chen Y.-Y. (2021). PAM4 rolling-shutter demodulation using a pixel-per-symbol labeling neural network for optical camera communications. Opt. Express.

[14] Jurado-Verdu C., Guerra V., Matus V., Rabadan J., Perez-Jimenez R. (2021). Convolutional autoencoder for exposure effects equalization and noise mitigation in optical camera communication. Opt. Express.

[15] Jurado-Verdu C., Guerra V., Rabadan J., Perez-Jimenez R. (2022). Deep learning for signal clock and exposure estimation in rolling shutter optical camera communication. Opt. Express.

[16] Chow C.-W., Liu Y., Yeh C.-H., Chang Y.-H., Lin Y.-S., Hsu K.-L., Liao X.-L., Lin K.-H. (2021). Display light panel and rolling shutter image sensor based optical camera communication using frame-averaging background removal and neural network. J. Light. Technol..

[17] Aranda J., Guerra V., Rabadan J., Perez-Jimenez R. (2024). Enhancing computational efficiency in event-based optical camera communication using N-pulse modulation. Electronics.

[18] Cubillo J.A., Guerra V., Azurdia-Meza C., Rabadan J., Perez-Jimenez R. (2025). Evaluation of pulse-shaped waveform modulation for event camera-based OCC systems. Electron. Lett..

[19] Ding X., Lu Q., Zhang Y., Li G., Gao X., Ye N., Niyato D., Yang K. (2026). Few-shot recognition and classification framework for jamming signal: A CGAN-based fusion CNN approach. IEEE Trans. Veh. Technol..

[20] Raspberry Pi Ltd. (2026). AI Camera Documentation. Raspberry Pi Documentation. https://www.raspberrypi.com/documentation/accessories/ai-camera.html.

[21] Sony Semiconductor Solutions Corporation (2026). IMX500 intelligent Vision Sensor. AITRIOS. https://www.aitrios.sony-semicon.com/edge-ai-devices/imx500.

[22] Zhang X., Dowhuszko A.A., Rêgo M., Fonseca P., Alves L.N., Hämäläinen J., Wichman R. (2026). Symbol rate maximization in rolling-shutter OCC: Design and implementation considerations. Proceedings of the ICC 2026—IEEE International Conference on Communications, Glasgow, UK, 24–28 May 2026.

[23] Bradley D., Roth G. (2007). Adaptive thresholding using the integral image. J. Graph. Tools.

[24] Ismail Fawaz H., Lucas B., Forestier G., Pelletier C., Schmidt D.F., Weber J., Webb G.I., Idoumghar L., Muller P.-A., Petitjean F. (2020). InceptionTime: Finding AlexNet for time series classification. Data Min. Knowl. Discov..

